# Reactive Case Detection for Malaria Elimination: Real-Life Experience from an Ongoing Program in Swaziland

**DOI:** 10.1371/journal.pone.0063830

**Published:** 2013-05-20

**Authors:** Hugh J. W. Sturrock, Joe M. Novotny, Simon Kunene, Sabelo Dlamini, Zulisile Zulu, Justin M. Cohen, Michelle S. Hsiang, Bryan Greenhouse, Roly D. Gosling

**Affiliations:** 1 Global Health Group, University of California San Francisco, San Francisco, California, United States of America; 2 Clinton Health Access Initiative, Mbabane, Swaziland; 3 Swaziland National Malaria Control Programme, Manzini, Swaziland; 4 Department of Pediatrics, University of California San Francisco, San Francisco, California, United States of America; 5 Department of Medicine, University of California San Francisco, San Francisco, California, United States of America; Tulane University School of Public Health and Tropical Medicine, United States of America

## Abstract

As countries move towards malaria elimination, methods to identify infections among populations who do not seek treatment are required. Reactive case detection, whereby individuals living in close proximity to passively detected cases are screened and treated, is one approach being used by a number of countries including Swaziland. An outstanding issue is establishing the epidemiologically and operationally optimal screening radius around each passively detected index case. Using data collected between December 2009 and June 2012 from reactive case detection (RACD) activities in Swaziland, we evaluated the effect of screening radius and other risk factors on the probability of detecting cases by reactive case detection. Using satellite imagery, we also evaluated the household coverage achieved during reactive case detection. Over the study period, 250 cases triggered RACD, which identified a further 74 cases, showing the value of RACD over passive surveillance alone. Results suggest that the odds of detecting a case within the household of the index case were significantly higher than in neighbouring households (odds ratio (OR) 13, 95% CI 3.1–54.4). Furthermore, cases were more likely to be detected when RACD was conducted within a week of the index presenting at a health facility (OR 8.7, 95% CI 1.1–66.4) and if the index household had not been sprayed with insecticide (OR sprayed vs not sprayed 0.11, 95% CI 0.03–0.46). The large number of households missed during RACD indicates that a 1 km screening radius may be impractical in such resource limited settings such as Swaziland. Future RACD in Swaziland could be made more effective by achieving high coverage amongst individuals located near to index cases and in areas where spraying has not been conducted. As well as allowing the programme to implement RACD more rapidly, this would help to more precisely define the optimal screening radius.

## Introduction

As countries move towards malaria elimination, methods to identify and treat infections among populations who do not seek treatment are required [1], [2], [3], [4]. Such individuals, many of whom are asymptomatic, may represent a substantial proportion of all infections and without treatment can remain infectious to mosquitoes [3], [5], [6]. As a result, relying on passive case detection (PCD) alone is unlikely to have the impact on the parasite population required to interrupt transmission. Active case detection (ACD), whereby individuals in a defined population are screened and treated for malaria parasites, targets this infectious pool regardless of whether or not infected individuals are symptomatic and is now recommended by WHO in moderate to low transmission settings [7]. Of note, in contrast to the days of the Global Malaria Eradication Program, these WHO guidelines include both febrile and non-febrile individuals in ACD activities.

One form of ACD is reactive case detection (RACD), whereby ACD is restricted to individuals living in close proximity to passively detected cases [4], [8]. This type of ACD takes advantage of the fact that infections are clustered spatially and temporally within transmission “hotspots” [9], [10], [11] and is being implemented widely in a number of eliminating countries including South Africa, Swaziland, Brazil and several countries in the Asia Pacific. In Zambia, researchers found that the prevalence of infection in household members of passively detected cases was 8.0% compared to 0.7% in randomly selected control households [8]. Similarly, in Peru, it was shown that the addition of RACD within a 100 m radius around households with previous history of infection yielded an incidence of confirmed malaria cases 4.3 times higher than passive case detection alone [12].

At the end of 2009, Swaziland’s National Malaria Control Programme (NMCP) initiated an active surveillance programme that aimed to conduct a case investigation on all confirmed cases at household level to determine source of infection as well as conduct RACD using a screening radius of 1 km around each passively detected case. Between October 2009 and November 2010, the active surveillance programme was focused primarily in the malaria at-risk Lubombo region in the eastern part of the country and human resources were limited to 3 officers who supported case investigation and RACD. The active surveillance programme has since been expanded to all regions of the country, however, RACD only takes place in receptive areas, determined by mapping the locations of historic cases and vector surveillance data. Currently, the NMCP’s surveillance team consists of a Chief Surveillance Officer (1), surveillance supervisors (2), and surveillance field agents (6). This team is supported by information technology officers (2) and a geographic information systems analyst who assist in the aggregation, validation, and analysis of surveillance data.

The NMCP developed a surveillance manual establishing a 1 km screening radius around an index case for RACD, which was determined based on estimated possible mosquito flight distance and an assessment of current NMCP capacity. However, it is unclear whether a 1 km screening radius is epidemiologically and operationally optimal in this setting. Using active surveillance data since the inception of RACD in Swaziland, this study examines the effect of screening radius, and other potential risk factors, on the probability of detecting rapid diagnostic test (RDT) positive individuals during RACD, in an effort to help inform the scales at which future RACD and other interventions are targeted.

## Methods

### Data Collection

Data were extracted from the Swaziland Malaria Surveillance Database System, which includes information gathered via active case investigation and detection on all confirmed malaria cases contacted and followed-up between December 2009 and June 2012. Details of this surveillance system employed are reported elsewhere [13]. Briefly, following detection of a confirmed case identified through health facilities (from here on termed “index case”), the NMCP is immediately alerted via Short Message Service (SMS). All cases are then traced back to their home where a case investigation is done and GPS coordinates are taken. Information collected during the investigation includes age, gender, bednet ownership, whether the household has been sprayed with insecticide, and recent travel history. A case is determined to be imported if the case reports travel to a high endemic area in the previous two weeks. A case is determined to be local if the case resides in, or travels to, a receptive area of Swaziland (determined by historical transmission records and historical vector surveillance records) and has not reported any travel to a high endemic area. Cases found in what are believed to not be receptive areas without any travel history are classified as cryptic or unknown. If the case is thought to be locally acquired by the investigator, all other household members and neighbouring households within a 1 km radius are to be screened using RDTs. Index cases were not retested due to the likelihood of testing positive by RDT to residual circulating antigens. If additional individuals are found to be positive by RDT (from here on termed a “secondary case”), they are treated using artemisinin-based combination therapies (ACTs). This reactive screening is also done for ‘high risk’ imported cases, at the discretion of the investigator, where either the local ecology is thought to be able to support transmission or if there are other individuals in the household who travelled with the index case.

### Data

All individuals included in the reactive case detection database between December 2009 and June 2012 were included in the analyses. These data do not include the index cases themselves. For the purpose of these analyses, coordinates for index cases lying outside Swaziland were re-coded as missing. Similarly, missing coordinate values were assigned to individuals more than 2 km from their corresponding index case as these were considered to be data entry errors. Distance to index case in kilometers was calculated from decimal degree coordinates using the gmt package in R 2.12 [14]. Guided by the total volume of passively detected cases, cases detected between January and May were classified as being in the high season and cases detected June to December as low season.

### Statistical Analysis

Logistic regression was used to model the probability of being a secondary case. The outcome of interest was, therefore, infection result by RDT in all individuals tested during RACD (excluding the index case). To avoid assuming linearity in the relationship between distance to the index case and being a secondary case, three distance classes were generated and explored as an explanatory variable in the regression model as an alternative to a continuous variable: within index household (i.e. with identical coordinates); <100 m from index household; and ≥100 m from household. The time between presentation of the index case at the health facility to the start of RACD was also explored as a linear, quadratic or categorical predictor (<1 week, 1–2 weeks and >2 weeks). All other covariates explored related to the index case as demographic information was not available on individuals screened during RACD. These included age of corresponding index case, both as a continuous and categorical variable (0–4, 5–9, 10–19, 20–39 and >40), gender of the index case, whether the index case was classified as local or imported, whether the index household had been recently sprayed by insecticide, whether the index case slept under a bednet the previous night and the season in which screening occurred. Information on these variables was available for the index cases but not the individuals screened during RACD. To account for correlation between individuals within each reactive case detection (i.e. all individuals relating to a single index case), a screening group level random effect was included in the model. Similarly, a second household level random effect was included to account for correlation between individuals within households. The final multivariate model was built in a forward stepwise fashion, including variables that remained significant at the 10% level in univariate regression analysis. Variables were retained if significant at the 5% level and where a likelihood ratio test at the 5% level suggested variable retention. Co-lineary between predictor variables was assessed by calculating the variance inflation factor values for each variable. Residual spatial autocorrelation between households was assessed by visual inspection of a semivariogram of household random effects. Statistical analyses were conducted using STATA 12.1 [15] and R 2.12 [14].

A second question addressed was whether the probability of detecting a case outside the index household was influenced by whether a secondary case was identified within the index household. If finding a case in the index household is a good proxy for ongoing local transmission, the programme can save time and resources by only conducting RACD in neighbouring households where an additional case is identified in the index household. In order to explore this issue, a second logistic regression model (model 2) was used to compare the probability of secondary cases being detected in non-index households in case detections where secondary cases were found in the index household compared to case detections where no secondary case was found in the index household. By necessity, only data from investigations where one or more individuals were screened in the index case household could be included in this analysis. The outcome of interest was RDT result of individuals in non-index households. For this analysis, detection of a secondary case in the index household was the only covariate explored. As for the first model, a screening group and household level random effect was included in the analysis.

### Coverage

In order to evaluate the coverage achieved by the surveillance programme, the number of households located within 1 km of 20 randomly selected index households was estimated manually by a single expert caller using satellite imagery (http://www.freemaptools.com/radius-around-point.htm). Spatially discrete individual structures, or clusters of smaller structures, were considered as a single household.

### Ethical Review

Review by the University of California Committee of Human Research was not required as this study involved the analyses of data generated through standard public health surveillance activities. Similarly, as the data were de-identified before analyses, according to the Health Insurance Portability and Accountability Act (HIPAA), patient information could be “…used and disclosed freely, without being subject to the Privacy Rule’s protections”. Individual consent was therefore not required.

## Results

### Cases

Over the study period, a total of 675 out of 1002 confirmed cases were investigated. 327 cases were not investigated due to loss to follow up, either because of inadequate recording of contact details or most often due to individuals leaving the country before an investigation could take place. Of those investigated, 344 (51%) were deemed imported based on travel history, 322 (47.7%) deemed local and 5 (0.7%) were undetermined. 250 of those investigated were deemed to live in receptive areas and triggered reactive case detection (Table 1). Of these 250 index cases, 3 had missing coordinates or coordinates lying outside Swaziland.

**Table 1:** Characteristics of the passively detected cases in Swaziland investigated between December 2009 and June 2012.

		Passively detected casesinvestigated (n = 671)	Investigated cases that triggered reactive case detection (n = 250)
Sex	Male	390 (58.1%)	151 (60.4%)
	Female	273 (40.7%)	97 (38.8%)
	Unknown	8 (1.2%)	2 (0.8%)
Mean age (range)		25.3 (1–81)	25.9 (1–81)
Season	High	524 (78.1%)	206 (82.4%)
	Low	147 (21.9%)	44 (17.6%)
Local/imported	Local	322 (48%)	163 (65.2%)
	Imported	344 (51.2%)	87 (34.8%)
	Unknown	5 (0.7%)	–
Owns a bednet	Yes	96 (14.3%)	59 (23.6%)
	No	575 (85.7%)	191 (76.4%)
House sprayed	Yes	137 (20.4%)	79 (31.6%)
	No	514 (76.6%)	169 (67.6%)
	Unknown	20 (3%)	2 (0.8%)

During the reactive case detection triggered by these index cases, 74 secondary cases were identified out of the 3671 individuals screened (2.02%) within 475 households. Of these households, 3 had no coordinates and a further 56 were located more than 2 km from their corresponding index case. Coordinates for these households were therefore recoded as missing before analyses.

### Screening Coverage and Timing

**Figure 1:**
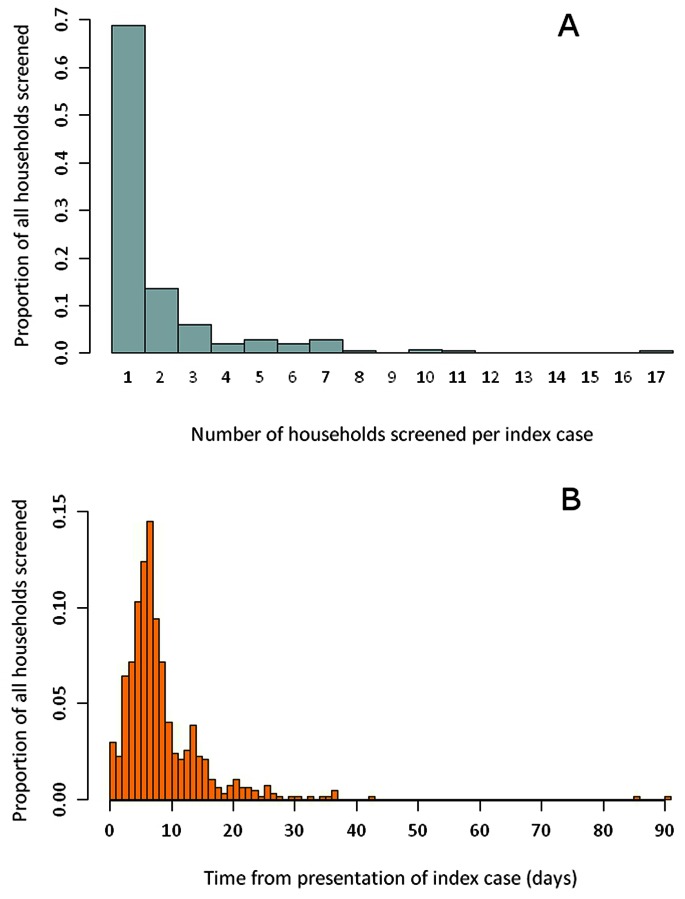
Household coverage and response time of RACD in Swaziland. A - Histogram of the number of households screened per index case (1 household indicates only the index household screened). B - Histogram of the time from presentation of the index case to the start of RACD.

46.6% of all individuals screened were within the same household as the index case. This tendency to screen just the index household is illustrated by a breakdown of the number of households surveyed per index case (Figure 1). 68.8% of the time, only the index household was screened. A median of 9 individuals (range 1–157) in a median of 1 household (range 1–17) were screened per index case.

Visual inspection of satellite imagery of structures surrounding an index case revealed that RACD is reaching only a small proportion of potential households within a 1 km radius (Table 2). 50% of individuals were screened within 7 days of the index case presenting at a health facility (range 0–91), with 87.3% screened within 14 days.

**Table 2:** Estimated number of households and actual number of households screened within 1 km of 20 randomly selected index households. Estimates of total number of households made using satellite imagery.

Index household	Number of households estimated within 1 km radius	Number of households screened within 1 km radius
1	27	1
2	24	6
3	35	1
4	>100	3
5	>100	1
6	48	1
7	>100	2
8	40	1
9	>100	1
10	37	1
11	46	3
12	34	1
13	28	2
14	34	1
15	38	2
16	80	3
17	43	1
18	7	1
19	>100	1
20	>100	3

### Risk Factors for being a Secondary Case

**Figure 2:**
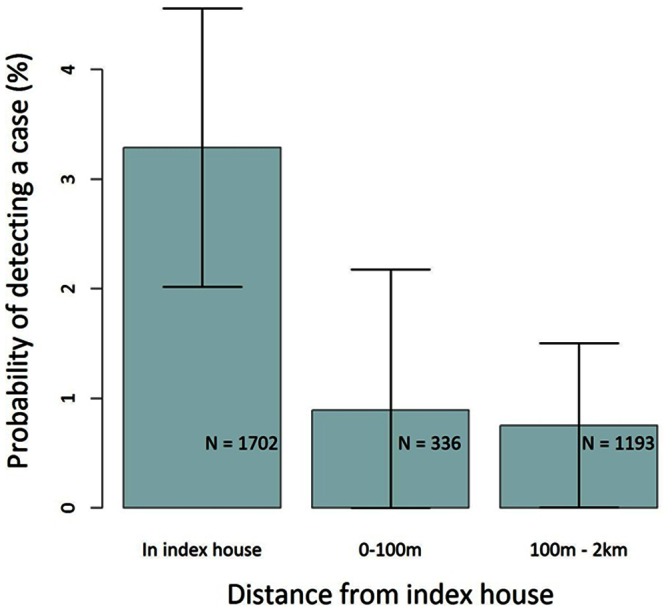
Probability of detecting a secondary case within different search distances from index households. **Error bars indicate the 95% confidence intervals (adjusted for intra-household correlation).**

The probability of detecting a case in the index household was 3.3% (95% CI 2.0%–4.6%), compared with 0.9% (0% –2.2%) and 0.8% (0% –1.5%) in households located 0–100 m and >100 m from the index house respectively (Figure 2). Results of univariate regression analyses of associations revealed that distance to index household was not linearly associated with detection of a case (p = 0.16). When distance to the index case was treated categorically, however, the odds of detecting a case within the index household were roughly 13 times higher (OR 13.2, 95% CI 3.2–53.6) than in households >100 m from the index household (Table 3). Odds of detecting a case within households 0–100 m from the index case were similar to those within households >100 m from the index house. Time from presentation of the case to RACD was not associated with detecting a case when included as a linear term (p = 0.24) and was borderline significant when included as a quadratic term (time: p = 0.07, time^2^: p = 0.05). When explored as a categorical variable, however, the odds of detecting a case within 1 week of presentation, were 12 times the odds of detecting a case >2 weeks from presentation (OR 12.2, 95% CI 1.37–108.8). There was little evidence that the odds of detecting a case 1–2 weeks from presentation were higher than >2 weeks from presentation (OR 6.7, 95%CI 0.8–56.0). Other factors significantly negatively associated with detecting a case included whether the index case was locally acquired (OR 0.3, 95% CI 0.1–0.81), whether the index house had been sprayed (OR 0.17, 95% CI 0.05–0.55) and whether the index owned a bednet (OR 0.17, 95%CI 0.04–0.74). Co-linearity was not suspected between these variables (all VIFs <2). Once included in a multivariable model, only distance to index, time from presentation and whether the household had been sprayed remained significant (Table 3). Semivariograms of household random effects suggested no evidence of residual spatial autocorrelation.

**Table 3:** Results of the univariate regression and the final multivariate regression model.

Variable		Univariate		Multivariate	
	Numbers positive/numbersexamined	Odds ratio	95% CI	Odds ratio	95% CI
>100 m from index household	9/1,193	1		1	
0–100 m from index household	3/336	1.76	0.2–15.1	2	0.22–17.7
In index household	56/1,702	13.1	3.2–53.6	13.0	3.1–54.5
Index owns does not own a bednet	69/2,921	1			
Index case owns a bednet	5/733	0.17	0.04–0.74		
Index house not sprayed	60/2,450	1		1	
Index house sprayed	13/1,151	0.17	0.05–0.55	0.11	0.03–0.46
Low season	13/586	1			
High season	61/3,068	1.24	0.46–3.36		
>2 weeks from presentation of index case	6/537	1			
1–2 weeks from presentation	33/1,341	6.7	0.8–56.0	4.3	0.61–29.9
<1 week from presentation	35/1,776	12.2	1.4–108.8	8.7	1.1–66.4
Imported	18/551	1			
Local	56/3,047	0.30	0.11–0.81		
Age		0.98	0.95–1.02		
Age category <5	5/163	1			
5–9	2/385	0.25	0.02–3.37		
10–19	17/860	0.54	0.08–3.75		
20–39	11/515	0.62	0.09–4.43		
>40	10/618	0.31	0.04–2.46		
Male	40/2,168	1			
Female	34/1,462	2.2	0.87–5.49		

Those terms with a 95% CI that span 1 were deemed non-significant. Terms were added in a forward stepwise fashion in the order in which they appear in the table.

Analysis of data from non-index households showed that the probability of being a case was not related to whether a case was identified in the index household (OR 0.36, 95% CI 0.03–3.54).

## Discussion

Reactive case detection is an important component of Swaziland’s malaria elimination campaign that allows detection of cases that would otherwise be missed by passive surveillance. It is, however, operationally demanding and resource intensive. Analyses show that the odds of detecting secondary cases during reactive surveys is significantly higher within the index household compared to other households located either<or >100 m from the index household. Furthermore, secondary cases were more likely to be detected when RACD was conducted within a week of the index presenting at a health facility and if the index household had not been sprayed with insecticide. Analyses using satellite imagery showed that a search radius of 1 km often includes an operationally challenging number of households to screen. Taken together, these results suggest that RACD is a useful method to detect infections that would be missed by passive surveillance. Furthermore, future RACD in Swaziland could be made more effective by ensuring surveillance teams are mobilized quickly and achieving high coverage amongst individuals located near to index cases and in areas where spraying has not been conducted.

The fact that cases appeared to be clustered within households is consistent with previous studies [16], [17], [18]. Evidence suggests that fine scale hotspots appear over all transmission settings, however these are more easily distinguished in lower transmission settings where the majority of individuals are free of malaria parasites [19]. Hotspots, which can be individual houses or clusters of houses, exist due to the presence of risk factors such as proximity to mosquito breeding sites, housing type and human behavioural and genetic factors [19]. Recent modeling work has shown targeting interventions at hotspots can lead to a disproportionately high impact on transmission [19]. Whilst this study supports the concept that reactive case detection may offer a method to identify these hotspots, it is possible that hotspots of purely asymptomatic infections can be missed using RACD due to the fact that symptomatic and asymptomatic malaria cases do not necessarily overlap spatially [9]. Whether this spatial incongruence between asymptomatic and symptomatic malaria exists in very low transmission settings is not clear and warrants further investigation. Other hotspots that might be missed include those with populations with low access to healthcare, situations where the index case is classified as a false negative and areas deemed unreceptive by the program. These issues may, in part, be overcome by the use of high resolution risk maps based on case data, that can aid the classifications of areas according to receptivity and guide implementation of proactive case detection in high risk areas underserved by the health system [20].

Data relating to the speed of investigation suggests that the programme is managing to respond to most cases within 1 week of presentation of the index case with only 12.7% of RACD conducted >14 days after presentation of the index case. The fact that the odds of detecting a case were much higher if RACD was conducted within a week of presentation of the index case may, in part, be due to the fact that a quick response allows asymptomatic cases to be detected before they become symptomatic and seek treatment. Early treatment of those infections ensures they have a limited opportunity to infect mosquitoes.

An interesting result was that the odds of finding secondary cases were lower when the index household was sprayed. Whilst data on whether neighbouring households had been sprayed was not available, it is likely that the index household acts as a marker for whether an area has been sprayed as spraying is usually targeted at predefined areas rather than individual houses. The apparent protective effect of a sprayed index household may therefore be due to the fact that neighbouring households had also been sprayed, lowering vector density in the area. It may also be that infections in sprayed areas are acquired outside the home during recreational or occupational activities.

It might have been expected that the odds of detecting a secondary case would be higher if the index case was classified as local. However, when accounting for distance to the index case and whether the household had been sprayed, there was no evidence to suggest an influence of index case classification on the probability of being a secondary case. This is most likely due to the fact that only imported cases that were deemed to be in receptive areas, or had travelled with others in the household, were selected for screening. Recording whether household members of imported cases travelled with the index case would help to assess whether these secondary infections were acquired whilst travelling, or whether local transmission has occurred. Alternatively, genotyping parasites may help to infer relatedness and the probability of a case being imported.

Analysis using satellite imagery showed that a 1 km screening radius includes a varying number of households, often with more than 30 per index case. In Swaziland, this presents a significant operational challenge which currently cannot be met with the available human resources. Indeed, analyses revealed that RACD was only being conducted in a small proportion of households within a 1 km radius of index households. While it was possible to estimate the number of households missed during RACD, information on individuals not present during RACD was not available. This information is now being collected and will help to establish whether a particular and potentially high risk demographic group is missed.

This study has a number of operationally relevant findings. Firstly, the significant clustering of infections within the household of an index case suggests that ensuring that all household members of index cases are screened should be a priority. That said, screening individuals in neighbouring households does appear to identify further cases which, given enough resources, should be considered given Swaziland’s goal of elimination. The distance at which screening is no longer able to identify cases could not be ascertained from this study as there appeared to be an equal probability of detecting cases in other households within the 2 km radius screened. This may be due to errors in the recording of coordinates and/or a small but consistent rate of false positives. Further studies using more accurate diagnostic methods, such as PCR, and ensuring a higher coverage of households around the index household are planned in Swaziland and should help to clarify this issue.

Secondly, surveillance teams need to be on the alert to enable a rapid response to passively detected cases, as the probability of detecting cases appeared to be higher within a week of an index case presenting. While such rapid mobilization may not always be possible during times of high case volume, focusing screening on immediate neighbours and in households that have not been sprayed, would help to streamline activities and allow the surveillance team to respond to new cases quicker.

Thirdly, passively detected cases appear to be useful for identifying at risk areas with insufficient coverage of IRS and ITNs. The apparent protective effect of IRS suggests that targeted focal spraying in areas around the index household may be of substantial value. As above, it is not clear over what scale such spraying should occur, and may well be dictated by the availability of resources, but should ideally cover at least the same area as the case detection.

Fourthly, these results support the concept of employing RACD on high risk imported cases as well as local cases. Defining these high risk cases is currently subjective and based on a combination of presence of historic cases in the area, as well as whether any other individuals have traveled with the index cases. Given the obvious importance of this group, a more standardized approach to classifying imported cases as high risk would be of value. Conducting reactive screening on a random sample of all imported cases may help to define these high risk cases.

Fifthly, results suggest that the decision to screen neighbouring households should not be made based on the finding (or not) of a secondary case in the index household, although this could be due to lack of statistical power. Whether this finding is consistent across ecological and epidemiological settings is not clear, but warrants further investigation as programmes that employ this decision criterion could miss a substantial proportion of infections.

Finally, this study also shows that freely available satellite imagery provides a practical and useful method to monitor and evaluate coverage achieved during reactive case detection. Indeed, following findings from this study, the National Malaria Control Programme in Swaziland now uses tablets with imagery visible offline to allow surveillance teams to estimate the number of households that should be screened and to navigate to those households. This should also allow the process of identifying households from satellite images to be validated on the ground. The use of tablets also allows GPS coordinates to be captured automatically, which should reduce errors. The ability to view satellite imagery offline may also be of use in a range of other settings such as household surveys, which currently rely on sketch maps to select households.

There are a number of limitations that should be highlighted. Firstly, due to the relatively small number of non-index households that were screened, it was not possible to establish whether there is a more subtle gradient of risk that exists around index households. Furthermore, no information on the number and characteristics of individuals that were missed was available, although this is important to ensure that high risk groups are captured. Future planned work will attempt to exhaustively screen individuals in households neighbouring cases and record information about those who are missing, allowing an exploration of these issues.

Secondly, the use of routinely collected data led to the possibility of bias and introduction of errors. Analyses assumed that the selection of neighbouring households by surveillance officers was random. There is a possibility that neighbouring households were in fact selected based on a suspicion of elevated risk in the household, such as the presence of individuals with a fever, or proximty to water. While we cannot quantify any such bias, should it exist, this would suggest that the difference in risk between index households and their neighbours would be even larger. Information on demographics and potential risk factors is now being collected on all individuals screened in RACD, as well as those missing during screening, in an effort to explore this issue further. Such information might also highlight risk factors which could be used to target RACD at certain groups. In addition to potential selection bias, it should be highlighted that a number of households screened were located >1 km from the index household. This could either be due to distance miscalculations during the reactive surveys, or due to errors in the recording of GPS coordinates. Whilst ensuring that future surveys and data entry should be conducted as accurately as possible, it still remains clear that there is a marked risk associated with being in the same household as the index case.

Thirdly, it should be noted that these data relied on results from RDTs which may miss infections in very low transmission settings. A study of submicroscopic infection revealed that contrary to traditional thought, the prevalence of submicroscopic infections appears to be highest in lower transmission settings [21]. Given that current RDTs are at best as sensitive as microscopy, this means that in a setting such as Swaziland using RDTs during RACD is likely to miss a number of infections [22]; Results from the latest MIS in Swaziland showed that RDTs missed the 2 individuals positive for *Plasmodium* by PCR and produced 3 false positives [23]. The ongoing development of PCR capacity in Swaziland will help to evaluate the performance of RDTs in this setting, but if relied on as the sole diagnostic would remove the ability to test and treat infections in a single field visit. PCR data would also allow a better understanding of the prevalence and spatial distribution of submicroscopic infections that go missed by RDTs. If submicroscopic infections cluster in close proximity to index cases, alternative strategies, such as targeted mass drug administration of households and immediate neighbours of passively detected cases, may be effective [24]. A similar approach has been suggested for schistosomiasis in low transmission settings, whereby individuals of households with school children who test positive are presumptively treated [25]. Development of loop-mediated isothermal amplification (LAMP), a diagnostic method with comparative sensitivity to PCR which can be used by non-expert technicians [26], is an exciting addition to the diagnostic toolkit, but requires further field evaluation particularly in low transmission settings. While microscopy is often used in Swaziland to confirm RDT results at health facilities, the use of point of care molecular tools would help to further limit both false negatives and false positives to ensure RACD takes place among populations at highest risk.

Fourthly, whilst this study supports the concept that RACD can be used to identify and target treatment at the pool of non-health-seeking infections, it was not possible to establish whether this resulted in a reduction in transmission. Such an issue will be addressed in ongoing and future work in Swaziland using molecular methods to investigate clustering of gametocytes around index cases. Interestingly, Stresman *et al.* (2010) found a gametocyte prevalence of 2.6% in households of passively detected cases versus 0% in randomly selected households [8]. Whilst the difference was not statistically significant, if shown to be a robust result, this has important implications for malaria elimination efforts, particularly for the choice of drugs used. Currently, the NMCP exclusively use ACTs to treat infected individuals with uncomplicated malaria, excluding pregnant women in their first trimester. If shown that gametocyte carriers can be identified using RACD, focused use of gametocidal drugs such as primaquine may have a significant impact on transmission. WHO recently recommended the use of a single 0.25 mg base/kg primaquine dose in all eliminating countries which have not yet adopted primaquine as a gametocytocide for falciparum malaria [27].

This analysis of Swaziland NMCP surveillance data demonstrates the ability of a functional RACD system to detect cases that would otherwise be missed by passive surveillance. Analyses suggest that the odds of detecting a case in the same household as passively detected cases are significantly higher compared to neighbouring households and in areas where the index household had not been sprayed with insecticide. Furthermore, cases were more likely to be detected if RACD was conducted within a week of the index cases presenting at a health facility. A 1 km screening radius appeared to be logistically challenging and may not be feasible in such resource limited settings such as Swaziland. Ensuring high coverage of households over a smaller screening radius and in areas that have not been sprayed would likely improve programme efficiency and responsiveness and would help to more precisely establish the optimum screening radius.
